# Feather Corticosterone Levels on Wintering Grounds Have No Carry-Over Effects on Breeding among Three Populations of Great Skuas (*Stercorarius skua*)

**DOI:** 10.1371/journal.pone.0100439

**Published:** 2014-06-25

**Authors:** Sophie Bourgeon, Eliza H. K. Leat, Ellen Magnusdóttir, Robert W. Furness, Hallvard Strøm, Aevar Petersen, Geir W. Gabrielsen, Sveinn Are Hanssen, Jan Ove Bustnes

**Affiliations:** 1 Norwegian Institute for Nature Research, Fram Centre, Tromsø, Norway; 2 Norwegian Polar Institute, Fram Centre, Tromsø, Norway; 3 College of Medical, Veterinary and Life Sciences, University of Glasgow, Glasgow, United Kingdom; 4 Iceland Institute of Natural History, Reykjavik, Iceland; University of Lausanne, Switzerland

## Abstract

Environmental conditions encountered by migratory seabirds in their wintering areas can shape their fitness. However, the underlying physiological mechanisms remain largely unknown as birds are relatively inaccessible during winter. To assess physiological condition during this period, we measured corticosterone concentrations in winter-grown primary feathers of female great skuas (*Stercorarius skua*) from three breeding colonies (Bjørnøya, Iceland, Shetland) with wintering areas identified from characteristic stable isotope signatures. We subsequently compared winter feather corticosterone levels between three wintering areas (Africa, Europe and America). Among females breeding in 2009, we found significant differences in feather corticosterone levels between wintering areas. Surprisingly, levels were significantly higher in Africa despite seemingly better local ecological factors (based on lower foraging effort). Moreover, contrary to our predictions, females sharing the same wintering grounds showed significant differences in feather corticosterone levels depending on their colony of origin suggesting that some skuas could be using suboptimal wintering areas. Among females wintering in Africa, Shetland females showed feather corticosterone levels on average 22% lower than Bjørnøya and Iceland females. Finally, the lack of significant relationships between winter feather corticosterone levels and any of the breeding phenology traits does not support the hypothesis of potential carry-over effects of winter feather corticosterone. Yet, the fitness consequences of elevated feather corticosterone levels remain to be determined.

## Introduction

Most seabirds breeding in temperate and Arctic regions are migratory [1] and spend most of the year away from their breeding colonies [2], [3]. For these species, the environmental conditions encountered on the wintering grounds have been shown to influence breeding performance, inducing profound fitness consequences (carry-over effects; [4], [5]). However, the underlying physiological mechanisms are largely unknown as birds are hardly accessible during winter. Lately, there has been tremendous technological progress to determine wintering ranges of individual migratory seabirds [3], [6], [7]. Corticosterone (CORT), the main stress hormone in birds [8], constitutes a physiological response of birds to changes in their environment. In american redstarts (*Setophaga ruticilla*), plasma corticosterone concentrations assessed during the non-breeding season appeared to be useful indicators of both physiological condition and winter habitat quality [9]. Assessing corticosterone concentrations in winter-grown feathers represents an alternative technique to assess physiological state during winter [10], [11], and relate it to life-history traits outside the wintering area. For example, high feather corticosterone levels on wintering areas were attributed to stressful conditions and population declines in European trans-Saharan migrant Egyptian vultures (*Neophron percnopterus*; [12]) and were negative predictors of future survival in wild house sparrows (*Passer domesticus*; [13]). Although the latter studies reported negative effects of elevated feather CORT levels on survival probabilities, they did not report effects on reproduction during the next breeding season. On the other hand, corticosterone levels measured in feathers grown months prior to egg-laying were shown to be positively associated with egg mass in atlantic puffins (*Fratercula arctica*; [14]). While feather CORT levels assessed in puffins were measured during the course of winter, no specific winter areas were identified.

Tracking data on great skuas (*Stercorarius skua*) that breed in three colonies in the northeast Atlantic (i.e., Bjørnøya, Iceland and Shetland) highlighted the use of geographically distinct wintering areas, also among individuals within a single breeding colony [15]. As previously outlined [16], [17], combining tracking data with stable isotope values of primary feathers successfully allowed to assign a larger sample size of untracked individuals to wintering areas [18] while avoiding any negative effect of loggers on stress levels of birds [19]. Namely, using breeding adult great skuas caught in three distinct colonies, we could determine that they wintered over three core areas i.e., continental shelf seas off America, Europe and Africa the previous winter [18]. None of the Shetland birds were reported to winter off America and while Bjørnøya and Iceland birds used the three wintering areas, they did so in different proportions [18]. Using the same winter grown feathers, we assessed corticosterone levels and subsequently compared individual levels between wintering area and breeding colonies. First, we predicted that feather CORT levels should vary between wintering areas reflecting different environmental conditions on the wintering grounds but should vary independently of the breeding colony subsequently used. To examine potential carry-over effects, we thereafter related corticosterone levels measured in feathers grown during winter to the breeding phenology of birds (i.e. body mass on return, egg laying date, initial clutch size, egg length and breeding success) the following breeding season. We expected that feather CORT levels should be suitable predictors of life-history traits measured during the subsequent breeding stage. However, based on the controversies previously reported [12]–[14], the nature of these relationships was hard to predict.

## Materials and Methods

Great skuas are large top predators with a female-biased sexual size dimorphism that breed in the North-East Atlantic. To avoid gender-specific differences in physiology and/or behaviour, 94 incubating females were caught in 2009 in three different colonies: Bjørnøya, Svalbard (74°21′N, 19°05′E) (N = 33), south-east Iceland (63°57′N, 16°24′W) (N = 32) and Foula, Shetland (60°08′N, 2°05′W) (N = 29) (see Bourgeon et al. [20] for a more detailed description of the sampling areas and techniques). Nests were followed from laying throughout chick rearing with laying date being calculated using hatching dates whenever unknown. The length of the eggs was measured to 0.1 mm accuracy using dial callipers. Females were caught on their nest while incubating using remote controlled noose traps. At each capture, body mass (±0.1 g) was recorded and blood was sampled from the brachial or tarsal vein using heparinised syringes, stored on ice and centrifuged within 2 h (5000 rpm); red blood cells were frozen and stored at −20°C and subsequently used to sex the birds (see below). The left eighth primary feather (third outermost) was cut and stored in individual sealed plastic bags at ambient temperature and was thereafter cut straight across perpendicular to the rachis to measure δ^13^C and δ^15^N stable isotopes (unpigmented vane of the proximal part of primary 8; see Leat et al. [18]) and feather corticosterone (distal part of primary 8; see below). Since great skuas start moulting their primary feathers (from the innermost outwards) in late autumn and through the winter [2], [21], primary 8 is expectedly grown around January when birds are still in the wintering areas, as indicated by the tracking data [15]. Both stable isotope and feather CORT values therefore represent those of the wintering area.

In Shetland, all procedures (i.e., remote controlled noose trapping, ringing, blood sampling and feather clipping) were carried out under licences from the Home Office (PPL 60/3835 awarded after ethical review of the protocol by The Home Office inspectorate) and the British Trust for Ornithology. In Bjørnøya, all procedures were carried out under permits from the Governor of Svalbard, Stavanger Museum and the Directorate for Nature Management. In Iceland, all procedures were carried out under the licence from the Icelandic Institute of Natural History, Reykjavik.

### a. Sexing, wintering area and feather corticosterone

Birds were sexed from red blood cells after DNA extraction and PCR amplification of CHD genes using primers 2550F (5′-GTT ACT GAT TCG TCT ACG AGA-3′)/2718 R (5′-ATT GAA ATG ATC CAG TGC TTG-3') [22] and 2550F/2757R (5′-AAT TCC CCT TTT ATT GAT CCA TC-3′) (R. Griffiths pers. comm.).

The wintering area of each bird was inferred using feather stable isotope values in conjunction with geolocator data [15] and continental shelf seas off America, Europe and Africa were identified as the three core wintering areas, any feather with a probability of geographical assignment lower than 0.95 being left unclassified [18]. In the current study, Bjørnøya birds mainly split between America and Europe (13 and 15 out of 33, respectively) but few wintered off Africa (5 out of 33). Half of the Iceland birds wintered off America (16 out of 32), whilst the remaining half equally split between Europe and Africa (8 out of 32 in each area). Birds from Shetland wintered in Europe or Africa (12 and 17 out of 29, respectively).

Feather corticosterone was measured using the distal 15 cm of the eighth primary and expressed in pg/mm of feather. Corticosterone was first extracted using a methanol-based extraction technique as reported by Bortolotti et al. [10]. Extracts were reconstituted in saline buffer, frozen at -20°C until subsequently assayed for corticosterone using an enzyme immunoassay kit (900-097, Assay Designs Inc., USA). We validated the use of this assay with feather samples by showing parallelism of serial dilutions of feather extracts (displacement curves) and the standard curve suggesting the absence of interfering substances in methanol extracts. The cross-reactivity of the assay is high with corticosterone (100%) but low with related steroids (e.g., progesterone: 1.7%; cortisol: 0.046%; β-estradiol: <0.03%). Feather extracts were measured in duplicate in 4 separate plates (using the same reagents) with intra- and inter-assay variability of 9.3% (N = 16) and 13.3% (N = 6), respectively. The concentration of corticosterone in feather samples was calculated by using a standard curve run on each plate.

### b. Statistical analyses

Statistical analyses were conducted using R version 2.15.2 [23]. Values are presented as means ± standard error (SE). Since feather corticosterone concentrations and all the reproductive phenology traits were normally distributed (Kolmogorov-Smirnov test, p>0.05), parametric tests were used. Type III general linear models (GLM) were first used to test for the effects of breeding colony (Bjørnøya, Iceland or Shetland) and wintering area (Africa, America or Europe; fixed factors, full factorial model) on feather CORT and life-history traits i.e., body mass, laying date, initial clutch size, A- and B-egg lengths and fledging success (dependent variables). Analyses of covariance (GLM) were subsequently used to assess the relationships between life-history traits (dependent variables) and feather CORT (independent variable) using breeding colony as a qualitative independent variable. The interaction between both predictors (i.e., feather CORT and colony) was omitted since none of the interactions were significant for any of the life-history traits (p>0.50 in all cases; data not shown). Data can be made freely available upon request.

## Results

**Figure 1:**
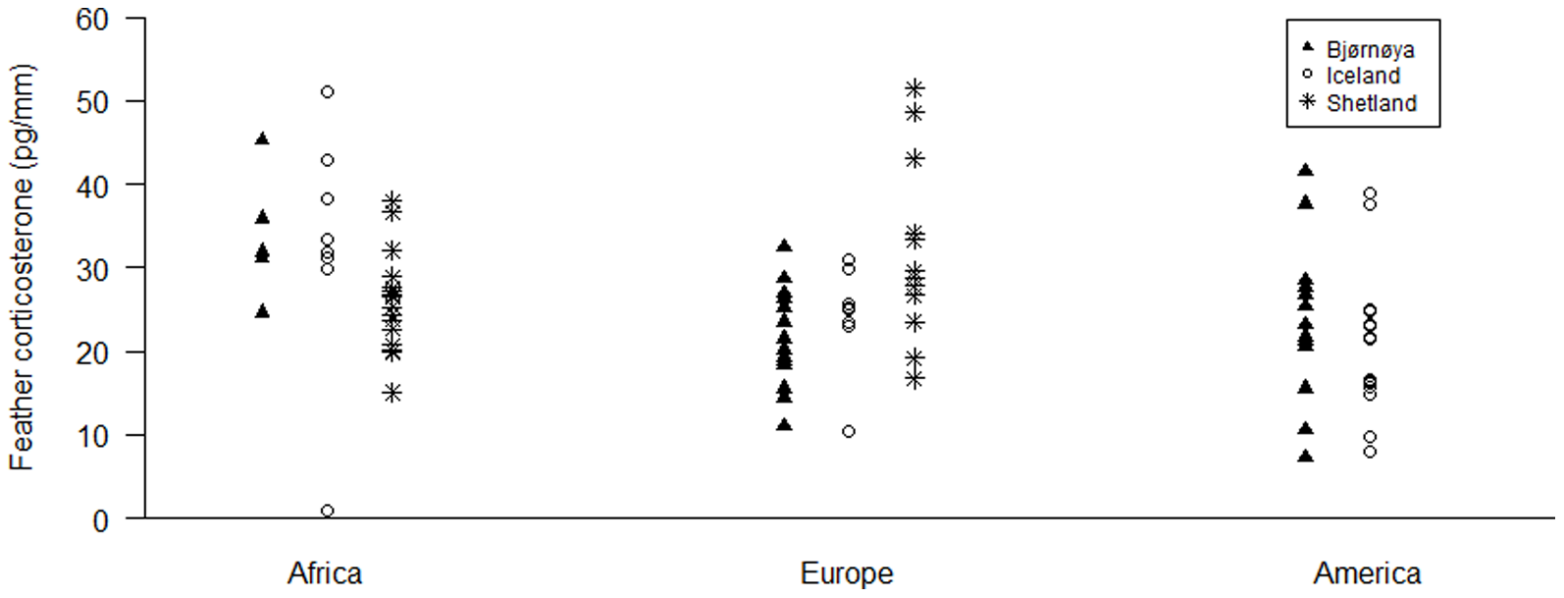
Individual feather corticosterone concentrations in winter grown primary feathers of great skuas (*Stercorarius skua*) breeding in three different colonies: Bjørnøya (black triangles), Iceland (white circles) and Shetland (black stars) in relation to their wintering quarters: Africa, Europe and America.

While there was no effect of breeding colony (GLM, F_2,86_ = 2.48, p = 0.09), wintering area significantly influenced feather CORT (GLM, F_2,86_ = 3.92, p = 0.02) which was significantly higher in Africa compared to America (28.89±1.75 pg/mm versus 22.14±1.65, respectively; Tukey's HSD *post hoc* test, p = 0.02), Europe being intermediate at 25.55±1.55 pg/mm (Tukey's HSD *post hoc* tests: Europe/America: p = 0.31; Europe/Africa: p = 0.32). Nevertheless, as indicated by the significant interaction between breeding colony and winter area (GLM, F_3,86_ = 5.15, p = 0.002), feather CORT did not vary consistently among colonies; Shetland birds that spent the winter in Africa showed the lowest feather CORT levels while birds from Iceland and Bjørnøya exhibited their highest levels whenever wintering in Africa compared to other areas (Table 1; Figure 1).

**Table 1:** Parameter estimates for type III general linear model (GLM) assessing the influence of wintering area (i.e., Africa, America and Europe) and breeding colony (i.e., Bjørnøya, Iceland and Shetland) on feather corticosterone levels of breeding great skua (*Stercorarius skua*).

					95% confidence interval	
**Parameter estimates**	Value	s.e.	t value	p value	lower	upper
Intercept	33.72	3.90	8.65	**<0.0001**	25.97	41.47
Colony (Iceland)	−1.31	4.97	−0.26	0.79	−11.19	8.57
Colony (Shetland)	−7.91	4.44	−1.78	0.08	−16.72	0.91
Winter (America)	−10.09	4.59	−2.20	**0.03**	−19.21	−0.97
Winter (Europe)	−12.56	4.50	−2.79	**<0.01**	−21.51	−3.61
Colony (Iceland) x Winter (America)	−1.39	5.94	−0.23	0.82	−13.20	10.42
Colony (Shetland) x Winter (America)	NA	NA	NA	NA	NA	NA
Colony (Iceland) x Winter (Europe)	4.40	6.27	0.70	0.48	−8.05	16.86
Colony (Shetland) x Winter (Europe)	18.66	5.57	3.35	**<0.01**	7.58	29.74

Parameter estimate values are given for the following factors: Colony: Bjørnøya; Winter: Africa. Numbers in bold indicate significant p-values (p<0.05). NA, not applicable; s.e., standard error.

We subsequently calculated two-way GLMs for each of the life-history traits (Table 2). There were no significant interactions between breeding colony and winter area for any of the traits tested (GLM, p>0.15; Table 2). While none of these traits were significantly influenced by the wintering area, four out of six significantly differed between colonies (Table 2). Thereafter, we ran analyses of covariance between each of the life-history traits and feather CORT using colony as a qualitative independent variable and found no significant relationship between feather CORT and any of the parameters (Table 3). These results suggest that winter feather CORT is not a good predictor for any of the breeding phenology traits assessed the following reproductive season.

**Table 2:** Influence of breeding colony (i.e., Bjørnøya, Iceland and Shetland) and wintering area (i.e., Africa, America and Europe) on reproductive phenology traits of breeding female great skuas (*Stercorarius skua*).

	Bjørnøya	Iceland	Shetland	Colony		Winter		Colony x Winter	
Dependent variables	N = 33	N = 32	N = 29	F value	p value	F value	p value	F value	p value
**Body mass (g)**	1433.48±11.33	1460.75±18.68	1416.15±14.19	0.09	0.92	1.78	0.17	0.10	0.96
**Laying date (julian date)**	168.69±1.06^a^	144.28±0.86^b^	148.93±1.17^c^	45.05	**<0.0001**	3.17	0.05	1.80	0.15
**Initial clutch size (eggs)**	1.57±0.09	2.00±0.00	1.93±0.05	2.16	0.12	1.32	0.27	0.44	0.72
**A-egg length (mm)**	68.52±0.56^a^	71.80±0.54^b^	70.86±0.66^b^	5.92	**<0.01**	1.87	0.16	1.61	0.19
**B-egg length (mm)**	67.13±0.83^a^	71.07±0.50^b^	68.69±0.41^a^	5.02	**<0.001**	0.83	0.44	0.60	0.62
**Chicks fledged**	0.12±0.06^a^	0.06±0.04^a^	0.59±0.13^b^	8.51	**<0.0001**	0.67	0.52	1.05	0.37

The F- and p-values were calculated using type III general linear models (GLM). Numbers in bold indicate significant p-values (p<0.05). Values (means ± standard errors) are reported for the 3 different breeding colonies. For each row reporting significant differences between colonies, lowercase letters (a–c) indicate a significant difference in means (Tukey's HSD *post hoc* tests).

**Table 3:** F-, p-values and parameter estimates for the analyses of covariance between life-history traits (dependent variables) of breeding female great skuas (*Stercorarius skua*) and winter feather corticosterone levels (pg/mm) and breeding colony (i.e., Bjørnøya, Iceland and Shetland) used as independent variables (additive model excluding the interaction).

INDEPENDENT VARIABLES	Feather corticosterone		Colony		Parameter estimates				
Dependent variables:	F value	p value	F value	p value		Value	s.e.	t value	p value
**1/Body mass (g)**	1.43	0.24	2.48	0.09	Intercept	1406.76	26.65	52.79	**<0.0001**
					Feather corticosterone	1.11	0.93	1.20	0.24
					Colony (Iceland)	26.60	20.66	1.29	0.20
					Colony (Shetland)	−22.64	22.28	−1.02	0.31
**2/Laying date (julian date)**	0.01	0.93	147.57	**<0.0001**	Intercept	168.55	1.87	90.09	**<0.0001**
					Feather corticosterone	0.01	0.06	0.09	0.93
					Colony (Iceland)	−24.42	1.49	−16.33	**<0.0001**
					Colony (Shetland)	−19.78	1.52	−13.05	**<0.0001**
**3/Initial clutch size (eggs)**	0.41	0.52	15.17	**<0.0001**	Intercept	1.63	0.11	15.32	**<0.0001**
**(CSini)**					Feather corticosterone	0.00	0.00	−0.64	0.52
					Colony (Iceland)	0.42	0.08	5.10	**<0.0001**
					Colony (Shetland)	0.37	0.09	4.24	**<0.0001**
**4/A-egg length (mm)**	3.68	0.06	9.50	**<0.0001**	Intercept	70.19	1.03	68.12	**<0.0001**
					Feather corticosterone	−0.07	0.04	−1.92	0.06
					Colony (Iceland)	3.30	0.80	4.14	**<0.0001**
					Colony (Shetland)	2.61	0.83	3.14	**<0.01**
**5/B-egg length (mm)**	2.66	0.11	12.72	**<0.0001**	Intercept	68.36	0.99	69.34	**<0.0001**
					Feather corticosterone	−0.05	0.04	−1.63	0.11
					Colony (Iceland)	4.02	0.81	4.94	**<0.0001**
					Colony (Shetland)	1.86	0.85	2.18	**0.03**
**6/Chicks fledged**	0.10	0.76	11.06	**<0.0001**	Intercept	0.08	0.15	0.57	0.57
					Feather corticosterone	0.00	0.01	0.31	0.76
					Colony (Iceland)	−0.06	0.11	−0.50	0.62
					Colony (Shetland)	0.46	0.12	3.89	**<0.0001**

Parameter estimate values are given for the following factor: Colony: Bjørnøya. Numbers in bold indicate significant p-values (p<0.05). s.e., standard error.

## Discussion

In migrating species, carry-over effects are described as the influences of environmental conditions encountered outside the breeding areas on the breeding performance the following year and/or fitness of individuals [4], [5], [24]. Nevertheless, the underlying physiological mechanisms remain unclear. It is believed that corticosterone is deposited into feathers as they grow [10], so assessing corticosterone in primary feathers collected on breeding birds likely represents corticosterone levels experienced during the previous winter. Among females breeding in 2009, we found significant differences in feather corticosterone levels between wintering areas frequented the previous winter. Namely, we found that feather CORT was significantly higher in birds wintering in Africa compared to America while levels measured in birds wintering in Europe did not significantly differ from those in Africa or America.

The differences in feather CORT could be attributed to different ecological factors such as local climatic conditions and/or food resources (abundance and quality) among the three wintering areas. Indeed, an increase in foraging activity can trigger increased plasma corticosterone levels [25], [26], and vice versa [27], [28]. However, the latter hypothesis is not in line with geolocator data collected on individual great skuas during the winter 2008–2009 that reported a significantly lower percentage of time spent in flight (used as a proxy for foraging activity) in birds wintering in Africa compared to the other areas [29]. Following the assumption that a low proportion of time spent foraging can indicate a favourable wintering ground (with either consistently good food supplies or dispersed but abundant preys), it was further concluded that, at least that year, Africa might have provided the best feeding opportunities of all the winter areas [29]. Another fact contradicting our theory of environmental conditions on the wintering grounds affecting feather CORT is the lack of consistency in feather CORT within breeding colonies. Indeed, contrary to our predictions and as indicated by the significant interaction between winter and breeding colony, females that shared the same wintering grounds showed significantly different concentrations of feather CORT depending on their breeding colony. This could be due to the distance travelled; while this scenario could apply to birds from Bjørnøya and Iceland which showed the highest feather corticosterone levels when travelling the longest distance (to Africa), it does not stand for the Shetland birds, which experienced higher feather corticosterone levels in Europe compared to Africa despite a shorter migration distance. Yet, feather CORT levels were the lowest in the winter areas where most of the females originating from each colony migrated (i.e., Africa for Shetland birds, Europe for Bjørnøya birds and America for Iceland birds). This could be the result of only experienced and/or higher quality females managing to reach their optimal winter area.

Regardless of the migration distance and environmental conditions on the winter areas, feather CORT might also be affected by persistent organic pollutants (POPs) whose concentrations and patterns were shown to vary between these winter areas [18], [30]. Nevertheless, while feather CORT were highest in Africa, POP exposure was globally lower in Africa compared to the other winter areas. Moreover, no clear relationships were identified between feather CORT levels and POP levels in breeding great skuas [20].

Ultimately, high corticosterone levels can be detrimental to immunity [31] and oxidative stress [32] and, due to their catabolic action, are shown to inhibit feather growth during moult [33]. While the latter effects are likely to have long-lasting effects during the following breeding season, the current study did not report any influence of wintering area on the breeding life-history traits assessed the following season. Moreover, unlike previous findings that reported positive effects of winter feather corticosterone levels on egg mass in atlantic puffins [14], our study failed at detecting any relationship between feather CORT and any of the breeding phenology traits. This could be due to the fact that all the birds used in our study i) survived the previous winter and ii) were successfully breeding the following summer, both being prerequisites for performing such a study. In addition, as suggested by our results, environmental conditions met on the breeding grounds probably constrain the outcome of breeding more than conditions on the wintering grounds. Nevertheless, it is noteworthy that feather CORT measured during winter might be more influenced by the previous breeding season rather than influencing the following breeding season. In black-legged kittiwakes (*Rissa tridactyla*), it was shown that the outcome of the breeding event (i.e. failure or success) influenced the date and place of migration while there was no evidence of any fitness difference between individuals wintering in different areas [34]. Since skuas seem to consistently use the same wintering areas from year to year [6], [15] regardless of the outcome of the previous breeding event, there could be a mismatch between the foraging abilities or physiological adaptations of the birds and the wintering areas further supporting the hypothesis that some skuas might use suboptimal wintering areas. Likewise, Eurasian spoonbills (*Platalea leucorodia leucorodia*) were shown to migrate to suboptimal wintering sites offering lower fitness prospects [35]. Winter feather corticosterone might therefore not be a good predictor of future breeding success in great skuas. Whether higher corticosterone concentrations during winter might have long-term effects on survival remain nonetheless unknown and future studies should investigate the matter.
